# Cardiac and renal biomarkers in recreational runners following a 21 km treadmill run

**DOI:** 10.1002/clc.23459

**Published:** 2020-09-03

**Authors:** Tee Joo Yeo, Lieng H. Ling, Carolyn Su Ping Lam, Jenny Pek Ching Chong, Oi Wah Liew, Zhen Long Teo, Lingli Gong, Arthur Mark Richards, Mark Y. Chan

**Affiliations:** ^1^ Cardiac Department National University Heart Centre Singapore Singapore Singapore; ^2^ Yong Loo Lin School of Medicine National University of Singapore Singapore Singapore; ^3^ Cardiovascular Research Institute National University Health System Singapore Singapore; ^4^ Department of Cardiology National Heart Centre Singapore Singapore Singapore; ^5^ Duke‐NUS Medical School Singapore Singapore

**Keywords:** amino‐terminal pro‐brain natriuretic peptide, biomarker, half‐marathon, high‐sensitivity troponin T, neutrophil gelatinase‐associated lipocalin, ventricular strain

## Abstract

**Background:**

Highly trained athletes running 42 km or more demonstrate elevated cardiac biomarkers, ventricular dysfunction, and decreased glomerular filtration rate (GFR). Whether similar changes occur in the much larger population of recreational runners following half‐marathon distance running is unclear.

**Hypothesis:**

Recreational runners exhibit changes in myocardial and renal biomarkers, including ventricular strain, after a half‐marathon treadmill run.

**Methods:**

10 recreational subjects (mean age 36.5 ± 6.5 years) ran 21 km on a treadmill (mean completion time 121.6 ± 16.1 minutes). Serum high‐sensitivity troponin T (hsTnT), amino‐terminal pro‐brain natriuretic peptide (NT‐proBNP), creatinine, and neutrophil gelatinase‐associated lipocalin (NGAL) were measured prior to, 1 hour post‐, and 24 hours post‐exercise. Pre‐ and post‐exercise echocardiograms were performed.

**Results:**

All biomarkers increased 1 hour post‐exercise: hsTnT by 8.5 ± 8.5 pg/ml (*P* < .05), NT‐ProBNP by 26.2 ± 22.8 pg/ml (*P* < .05) and NGAL by 29.5 ± 37.7 ng/ml (*P*=NS). By 24 hours post‐run, these biomarkers declined toward baseline levels. Right ventricle (RV) free wall and left ventricle global longitudinal strain decreased by 5.5% and 1.8%, respectively (*P* < .001). Changes in NGAL correlated well with changes in serum creatinine (R = 0.79, *P* < .01) and GFR (R = −0.73, *P* < .05). Faster 21 km completion times, and a larger reduction in post‐exercise RV strain, were associated with higher NGAL levels: (R = −0.75, *P* = .01) and (R = 0.66, *P* < .05), respectively.

**Conclusion:**

A 21 km run in recreational runners is associated with transient ventricular stunning and reversible changes in myocardial and renal biomarkers. Whether repeated bouts of similar activity contributes to chronic cardiac or kidney dysfunction deserves further evaluation.

## 1 INTRODUCTION

Prolonged endurance exercise has been associated with elevation of cardiac troponins, amino‐terminal pro‐B‐type natriuretic peptide (NTProBNP),^1^, ^2^, ^3^ biventricular dysfunction,^4^, ^5^, ^6^ and acute kidney injury (AKI).^7^, ^8^ While the advantages of physical activity are well‐accepted, these observations have raised concerns of the loss of health benefits and even potential harmful effects‐such as atrial fibrillation, atherosclerosis, and myocardial fibrosis‐with excessive exercise.^9^, ^10^

Most studies have focused on highly trained athletes participating in long distance endurance activities. However, participants of the full marathon distance (42.125 km) and above are a minority compared with those of shorter distance events. Over the last 15 years, the number of half‐marathon (21 km) participants globally has increased more than 5‐fold from under 500 000 in 2003 to more than 2 million in 2018. The 21 km distance had twice the number of participants as the full marathon distance and was second only to the number of 5 km runners (2.9 million).^11^ Despite the large numbers of recreational endurance runners, there are limited data on the physiological effects of half‐marathon distance running in this population. Given the increasing importance of half‐marathon distance running, it is important to better understand the physiology of this distance among non‐elite athletes.

In this pilot study, we investigated changes in peripheral blood biomarkers of myocardial injury, haemodynamic stress, and renal injury with right and left ventricular strain in recreational runners completing a 21 km treadmill run.

## 2 METHODS

### 2.1 Study population and protocol

Study participants were recruited from a local recreational running club. Eligible subjects were recreational runners 21 years or older with a weekly training distance of under 50 km.^12^ In contrast, highly trained endurance athletes have average weekly training distances of 120 to 200 km.^13^ Potential participants were screened by completing a comprehensive questionnaire. Individuals with any history of cigarette smoking, diabetes, hypertension, hyperlipidemia, consumption of any heart rate lowering medication, and those with a family history of sudden cardiac death were excluded from this study.

Training history was ascertained for all participants including average running distance per week, number of years of running, and number of half‐ and full‐marathons completed. Measurements of height, weight, peripheral blood pressure, and resting heart rate were obtained prior to the run. Holter monitors were attached to all runners for the duration of the run to track their exercise heart rates. Maximum heart rate for each participant was calculated by subtracting their age from 220. Level of intensity was then calculated by dividing average heart rate during activity by maximum heart rate. Based on the American College of Sports Medicine (ACSM) guidelines, a range of 30% to 49% of maximum heart rate corresponds to “light” intensity, 50% to 69% to “moderate” and 70% to 89% to “hard”.^14^

All subjects completed an indoor 21 km run at their own pace within an air‐conditioned gymnasium on a commercial grade treadmill (Precor C946i, Woodinville, WA) with full manual settings. Isotonic beverages and plain water were made available throughout the run and participants were encouraged to hydrate liberally. Fluid intake of the subjects was not monitored. Transthoracic echocardiography (TTE) was performed pre‐run and within an hour of completing the 21 km run. Peripheral blood samples were obtained pre‐run, within 1 hour post‐run and within 24 hours post‐run.

The protocol was approved by the local institutional review board ethics committee and all subjects gave written informed consent prior to study participation.

### 2.2 Biochemical studies

Serum creatinine was measured by the Enzymatic Creatinine 2 method using the Advia 2400 analyzer (Siemens Healthcare Diagnostics, Erlangen, Germany). The reference range for this assay is 9‐2652 umol/L, and upper limit of normal is 125 umol/L and 90 umol/L in males and females, respectively. Estimated glomerular filtration rate (GFR) was then calculated using the Modification of Diet in Renal Disease (MDRD) equation.^15^

High‐sensitivity troponin T (hsTnT) and NT‐proBNP were measured using electrochemiluminescence immunoassays on a cobas e411 analyzer (Roche Diagnostics GmbH, Mannheim, Germany). For hsTnT, the 99th percentile for normal subjects is 14 pg/ml. For NT‐ProBNP, the upper limit of normal in subjects 75 years of age and below is considered to be 125 pg/ml.

Serum neutrophil gelatinase‐associated lipocalin (NGAL) was quantified using enzyme‐linked immunosorbent assay with the Quantikine Human Lipocalin‐2 immunoassay (R & D Systems Inc, Minneapolis, MN) and absorbance values measured on the Enspire Microplate Reader (Perkin Elmer, Waltham, MA). The normal range for healthy individuals is 42‐177 ng/ml (mean 119 ng/ml ± 35.6).

### 2.3 Echocardiography

Subjects underwent M‐mode, two‐dimensional (2D) and doppler TTE using a Vivid 7 Dimension ultrasound system (GE Healthcare, Milwaukee, WI) equipped with a 2.5 MHz probe, in accordance with American Society of Echocardiography (ASE) guidelines.^16^, ^17^ These were analyzed by a neutral physician blinded to the results of the endurance exercise.

Left ventricular (LV) ejection fraction and stroke volume were measured using the biplane method of disks. Fractional shortening was calculated using the difference in LV end‐diastolic and end‐systolic diameters via M‐mode. Pulsed wave tissue doppler imaging was performed to obtain peak early (e') and late (a') myocardial velocities and the ratio of early diastolic transmitral flow velocity to e' (E/e').

Indices of myocardial deformation obtained by speckle‐tracking analysis using the EchoPac software (Version 11.1.8, GE Healthcare, Horten, Norway). LV global longitudinal strain (GLS) was obtained as the average segmental value based on three apical imaging planes.

Right ventricular (RV) free wall strain was assessed along the length of the free wall from apical 4‐chamber images. Other measures of RV systolic function that is, 2D fractional area change (FAC), tricuspid annular plane systolic excursion (TAPSE) and myocardial performance index (via pulsed tissue Doppler) were also obtained.^18^ Right atrial pressure (RAP) was calculated.^19^, ^20^ Inferior vena caval dimension was measured during expiration, just proximal to its junction with the hepatic vein.^18^ Mean pulmonary artery pressure (mPAP) was estimated using Mahan's regression equation.^20^

### 2.4 Statistical analysis

Data for continuous variables are presented as mean ± SD. Repeated measures analysis of variance (ANOVA) was used to compare changes in the biomarkers for all 3 time points (before the 21 km run, 1‐hour post‐run and 24‐hours post ‐run. Spearman's rank correlation coefficient was used to calculate the relationship between two continuous variables. Statistical analyses were performed with the use of STATA version 11.1 (Statacorp, College Station, TX). A 2‐sided P value of <.05 was considered statistically significant.

## 3 RESULTS

### 3.1 Baseline parameters (Table 1)

**TABLE 1:** Baseline characteristics and vital signs of participants

	Total	Males (n = 6)	Females (n = 4)
**Age (years)**	36.5 ± 6.5	38.2 ± 6.4	34 ± 6.8
**Women (%)**	40		
**Body mass index (kg/m** ^**2**^ **)**	21.9 ± 1.7	22.8 ± 1.4	20.6 ± 1.1
**Systolic blood pressure (mmHg)**	120 ± 11.6	136 ± 12.7	113 ± 7.4
**Diastolic blood pressure (mmHg)**	72.8 ± 8.4	83.8 ± 6.1	73.8 ± 7.1
**Heart rate (beats per min)**	59 ± 6.7	74 ± 8.6	64 ± 5
**Weekly running distance (km)**	29 ± 9.1	25 ± 8.9	35 ± 5.8
**Running experience (years)**	4.25 ± 3.1	3.3 ± 1.5	5.6 ± 4.6
**Run completion time (min)**	121.6 ± 16.1	122.7 ± 20	120 ± 10.1

The study population consisted of 10 subjects (40% female) with mean age of 36.5 ± 6.5 years and mean body mass index of 21.9 ± 1.7 kg/m^2^. Participants had 4.25 ± 3.1 years of running experience, and ran 29 ± 9.1 km weekly. Mean pre‐exercise values for systolic and diastolic blood pressure and heart rate were 120 ± 11.6 mmHg, 72.8 ± 8.4 mmHg and 59 ± 6.7 beats per minute respectively. All participants were free of modifiable cardiovascular risk factors.

Participants completed the 21 km treadmill run in an average time of 121.6 ± 16.1 minutes (122.7 ± 20 minutes for male subjects, compared with 120 ± 10.1 minutes for females). These timings correspond to the 60 to 70th percentile and 30 to 40th percentile for recreational male and female runners respectively, and 40 to 50th percentile overall.^21^ Average heart rate during the run was 149.8 ± 11.5 bpm. This translated to 81.6 ± 5.1% of their age‐predicted maximum heart rate.

### 3.2 Biomarkers (Figure 1)

**FIGURE 1:**
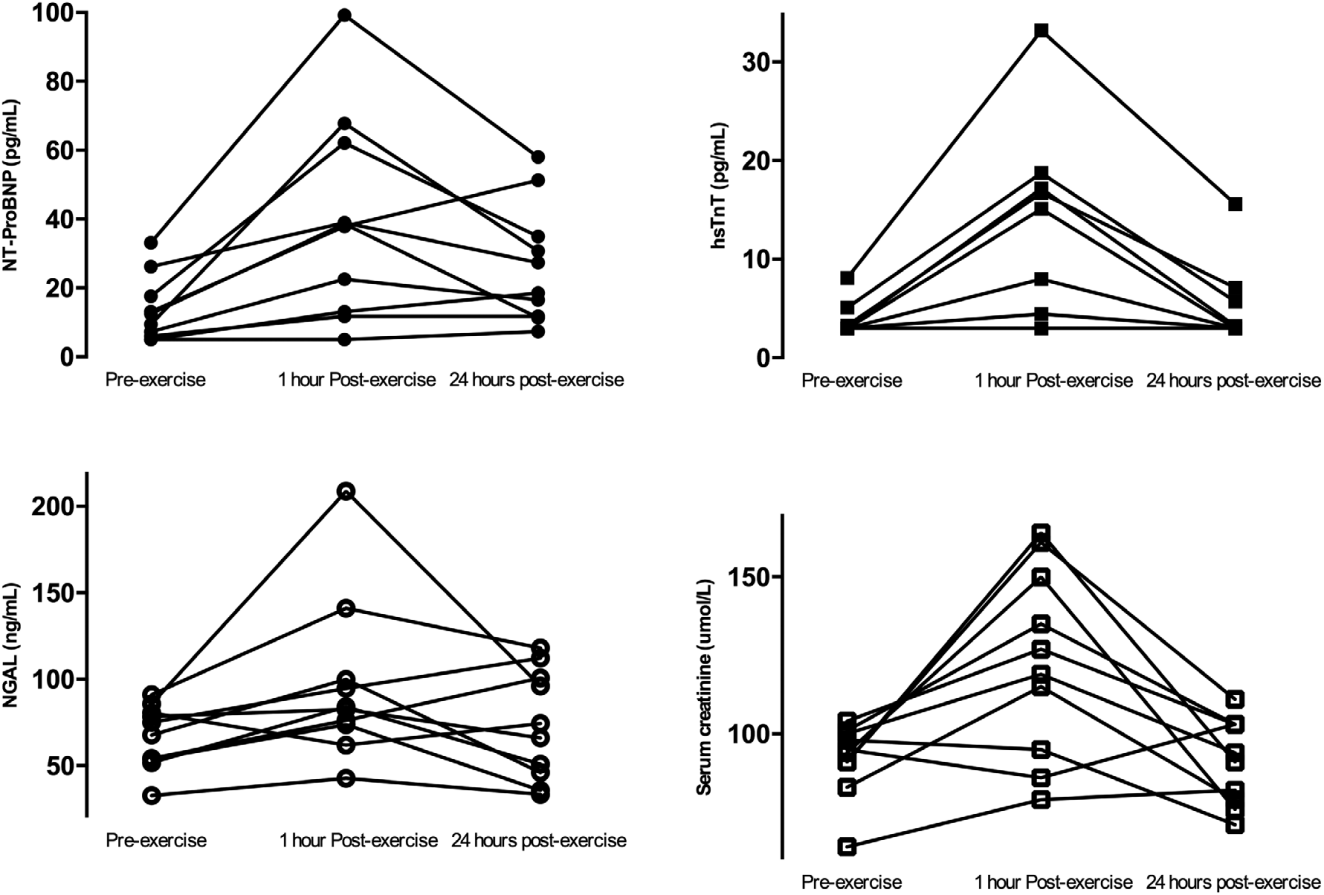
Changes in biomarkers prior to, 1 hour after, and 24 hours after a 21 km run

Baseline hsTnT levels ranged from 3 to 8 pg/ml. Within 1 hour of completing the run, hsTnT increased to 12.2 ± 9.8 pg/ml (*P* = .01) and returned toward baseline 24 hours post‐run (mean 5.0 ± 4.0 pg/ml, *P* < .01). In 5 runners (2 males and 3 females), the 1‐hour post‐run hsTnT was elevated beyond 14 pg/ml. There was no correlation between running intensity or run duration and hsTnT level.

Mean NT‐proBNP at baseline was 13.5 ± 10 pg/ml and increased to 39.7 ± 29 pg/ml (*P* < .01) within 1 hour post‐run. In no subject was the upper limit of normal exceeded. As with hsTnT, levels of NT‐proBNP declined at 24 hours post‐run to 26.8 ± 17 pg/ml (*P* = .06). There was no correlation between NT‐proBNP and hsTnT.

Mean NGAL pre‐run was 67.1 ± 18.4 ng/ml and increased 1 hour post‐run to 96.6 ± 47.1 ng/ml (*P* < .05). At 24 hours post‐run, mean NGAL dropped to 73.4 ± 31.8 ng/ml (*P* = .1), consistent with the other biomarkers. At baseline, serum creatinine was 92.1 ± 11.6 umol/L and increased to 123.1 ± 30.2 umol/L (*P* < .01). Pre‐run estimated GFR (73 ± 10 ml/min/1.73m^2^) declined by 25% to 55 ± 18 ml/min/1.73m^2^ (*P* < .01) at 1 hour post‐run. NGAL levels correlated with both serum creatinine (R = 0.79, *P* < .01) and GFR (R = −0.73, *P* < .05). Shorter 21 km completion time was associated with higher NGAL 24 hour post‐exercise (R = −0.75, *P* = .01).

### 3.3 Echocardiography (Table 2)

**TABLE 2:** Echocardiographic analysis prior to and 1 hour after a 21 km run

	Pre‐exercise	1 hr post‐exercise	*P*
**Right ventricular analysis**			
**Fractional area change (%)**	52.7 ± 7.4	41.2 ± 7.7	.002
**Tricuspid annular plane systolic excursion (mm)**	20.2 ± 1.9	17 ± 3.2	.006
**Tricuspid annulus systolic velocity (S') (cm/s)**	12.6 ± 2.1	11.7 ± 2.6	.235
**Myocardial performance index**	0.54 ± 0.14	0.58 ± 0.11	.45
**Mean pulmonary arterial pressure (mmHg)**	9.7 ± 7.4	16.9 ± 6.6	.033
**Tricuspid annulus E/e'**	4.49 ±0.75	4.54 ± 1.09	.92
**Right ventricular diastolic area (cm** ^**2**^ **)**	17.8 ± 2.8	15.5 ± 2.9	.08
**Hepatic vein systolic velocity (cm/s)**	37 ± 13.7	34.6 ± 13.3	.7
**Inferior vena cava dimension (cm)**	1.65 ± 0.26	1.34 ± 0.49	.1
**Mean right atrial pressure (mmHg)**	7.1 ± 1.3	6.6 ± 1.7	.52
**Left ventricular analysis**			
**Fractional shortening (%)**	37.6 ± 4.6	34.4 ± 4.2	.045
**Ejection fraction (%)**	63.5 ± 2.1	62.6 ± 2.8	.484
**E/A ratio**	1.82 ± 0.4	1.09 ± 0.34	.001
**Mean of septal & lateral E/e'**	6.7 ± 1.2	5.9 ± 1.1	.025
**Mean of septal & lateral e'**	11.7 ± 1.4	10.3 ± 1.2	.002
**Internal diameter in diastole (mm)**	49.2 ± 2.6	47.5 ± 3.4	.094
**Stroke volume (ml)**	69.8 ± 13.4	64.2 ± 13.0	.044
**End diastolic volume (ml)**	111.8 ± 22.8	103.1 ± 20.6	.052
**Cardiac index (L/min/m** ^**2**^ **)**	2.4 ± 0.3	2.7 ± 0.5	.135
**Strain analysis**			
**Right ventricular free wall strain (%)**	−21.75 ± 3.34	−16.21 ± 4.39	<.001
**Left ventricular global longitudinal strain (%)**	−17.79 ± 2.4	−15.99 ± 2.24	<.001

Baseline echocardiographic parameters were within normal limits in all subjects. Post‐exercise, LV ejection fraction, end diastolic volume, and cardiac index were unchanged, whereas LV stroke volume and fractional shortening decreased, falling by 5.7 ± 2.4 ml (*P* < .05) and 3.2 ± 1.4% (*P* = .05), respectively. The septal E/e' ratio remained stable pre‐ and post‐exercise, while the lateral LV E/e' ratio declined by 1.1 ± 0.8 (*P* < .01). At 1 hour post‐run, RV FAC decreased by 11.5 ± 2.6% (*P* < .01), TAPSE decreased by 3.2 ± 2.8 mm (*P* < .01) and mPAP increased by 7.2 ± 2.9 mmHg (*P* < .05). RV annular systolic velocity and RV myocardial performance index did not change significantly. RAP, IVC dimension, and other surrogate measures of RV filling, including RV diastolic area, tricuspid E/e' and hepatic vein systolic velocity, showed no statistically significant differences before and after exercise.

### 3.4 Strain imaging (Table 2 and Figure 2)

**FIGURE 2:**
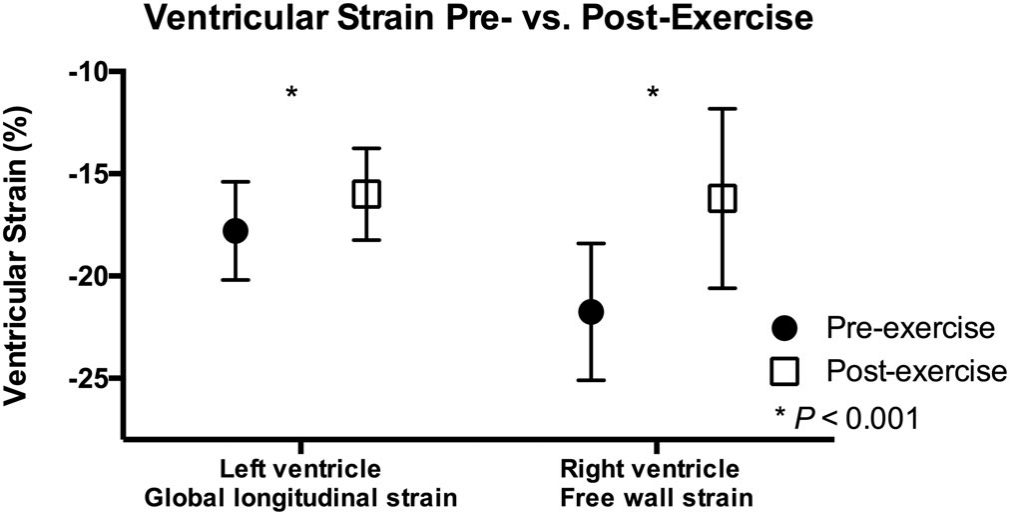
Ventricular strain prior to and 1 hour after a 21 km run

Biventricular strain showed statistically significant reductions following endurance exercise. There was no difference in the magnitude of reduction in LV GLS between runners with post‐run hsTnT above versus below the 99th percentile. The percentage reduction in RV free wall strain showed a positive relationship with peak (R = 0.64, *P* = .05), as well as 24‐hour post‐exercise NGAL (R = 0.66, *P* < .05), but did not correlate with running time or the extent of increase in PASP. In the four subjects who had higher NT‐proBNP level at 24 hours than at 1 hour post‐exercise, there was a greater reduction in RV strain compared with those in whom NT‐proBNP declined (5.9 vs 2.7%, *P* = .05).

## 4 DISCUSSION

In this study, we assessed the effects of exercise in recreational runners completing a 21 km run with continuous access to oral rehydration in a controlled, air‐conditioned indoor environment. Highly sensitive biomarkers were used to determine if an endurance run of 21 km was associated with potential myocardial or renal injury. In these individuals, a 21 km treadmill run led to: (a) a transient and reversible elevation of hsTnT and NT‐proBNP, (b) increased NGAL and reversible reduction in estimated GFR, and (c) myocardial stunning as evidenced by reduced biventricular strain. In addition, NGAL showed good correlation with the degree of ventricular stunning.

A meta‐analysis of 26 studies showed that 47% of endurance athletes had elevation in cardiac troponin post‐exercise.^1^ However, significant knowledge gaps remain. The majority of studies pooled in this meta‐analysis examined endurance activity exceeding 2 hours and did not include non‐highly trained recreational runners. Moreover, older generation troponin assays were used instead of hsTnT. Our findings suggest that distance running below 42 km in recreational runners can also elevate cardiac biomarkers. Studies involving marathon‐distance running and beyond suggest exercise‐induced cardiac troponin elevation is most likely due to diffusion of cardiac troponin from the intra‐ to extra‐cellular space, and non‐pathological in nature.^2^, ^6^ In our study, half of the runners showed post‐run elevations of hsTnT above the 99th percentile without correlation with gender, running intensity, NT‐proBNP levels, and magnitude of reduction in ventricular strain. In contrast, within a cohort of middle‐aged individuals who participated in 8.3 hours of endurance walking, those with post‐exercise troponin elevation beyond the upper reference limit were almost 2.5 times more likely to experience adverse cardiovascular events over 43 months of follow‐up compared with those without troponin elevation.^22^ Although the participant demographics and activity level differs from our study, it reinforces the uncertainty surrounding exercise‐related troponin elevation and highlights the potential long‐term prognostic utility of hsTnT.

Independent studies utilizing magnetic resonance imaging in competitive lifelong athletes have shown an association between long‐term endurance activity and the presence of myocardial fibrosis, with reported prevalence rates as high as 17%.^23^, ^24^, ^25^ Conversely, other studies encompassing individuals with a broad spectrum of physical activity levels have shown minimal late gadolinium enhancement.^26^, ^27^ These conflicting data suggest endurance exercise and myocardial scarring remains a highly controversial topic.

Our study showed a statistically significant reduction in LV GLS, in keeping with previous work on athletes participating in prolonged endurance activity.^28^, ^29^ La Gerche et al. showed that in well‐trained athletes, endurance exercise causes RV dysfunction, manifested as reductions in RV ejection fraction, FAC, TAPSE, and free wall strain.^4^ Similar changes in RV parameters were demonstrable in our study. In addition, four runners showed persistently elevated NT‐proBNP at 24 hours that correlated with greater reduction in RV strain, although there was no other association with age, running experience or hsTnT to suggest different levels of vulnerability to RV dysfunction. Whether post‐endurance exercise LV or RV stunning persists and contributes to exercise‐induced cardiomyopathy in the long term in these recreational runners should be addressed in prospective longitudinal studies.

Although fluid intake was not restricted throughout the run, dehydration and reduced preload could account for the right ventricular changes. While fluid balance was not directly quantified, comparison of multiple surrogate measures of RAP and RV filling pressure showed no statistically significant differences pre‐ and post‐run. Moreoever, LV dimensions (both internal diameter and end‐diastolic volume) did not change significantly post‐run. Taken together, this suggests that the observed reduction in RV strain was predominantly related to transient myocardial stunning rather than altered load.

In our study, pre‐run estimated GFR declined by 25% at 1 hour post‐run, fulfilling stage 1 of the RIFLE (acronym of Risk, Injury, Failure, Loss, and End‐stage kidney disease) classification system for AKI.^30^ This occurred despite an air‐conditioned indoor environment and full access to oral hydration throughout the run. The accurate estimation of GFR from serum creatinine requires a steady state, which is not necessarily the case in our study. However, estimated GFR based on the non‐steady state serum creatinine overestimates measured GFR,^31^ hence GFR immediately post‐run may have been even lower than estimated. Moreover, measured NGAL correlated with both serum creatinine as well as estimated GFR.

NGAL is a novel highly sensitive biomarker for AKI.^32^ Evidence for renal impairment following endurance exercise comes from the studies by McCullough et al.^7^ and Lippi et al.,^33^ who demonstrated elevations of NGAL in endurance athletes who completed a 42 km marathon and a 60 km ultramarathon, respectively. Our study shows that transient increases in NGAL may also occur with running distances below 42 km. Although NGAL in all runners did not exceed the upper reference limit, the correlation between run completion time and NGAL suggests that more intense exercise may increase the risk of renal dysfunction. While McCoullough et al. demonstrated a 5‐fold elevation in urinary NGAL following marathon running, they could not show a relationship between marathon time, a proxy of effort, and the rise in renal markers. The more controlled environmental setting with continuous access to oral rehydration, under which our study was conducted, may possibly account for the difference in findings. Christensen et al. studied 10 Tamahumara runners following a 78 km race and measured copeptin as a surrogate for renal function. The rise and fall of copeptin post‐race mirrored that of NGAL in our study, and the authors attributed this to exercise‐associated systemic plasma volume depletion.^34^ While this is a feasible explanation, we showed that the change in NGAL correlated well with the percentage drop in RV strain (non‐load related). Nonetheless, it would be premature to suggest a pathophysiological interaction between the heart and kidneys based on the mild and transient changes in our study. Further studies utilizing direct measurement of plasma volumes as well as both urinary and blood biomarkers will be necessary to elucidate any potential heart‐kidney relationship during endurance activity.

Limitations of our study included its small sample size, making generalisability an issue. Additionally, cardiac magnetic resonance, the current imaging gold standard, was not utilized for more accurate assessment of cardiac volumes and function due to operational constraints. However, each subject served as their own control, and any inaccuracies in echocardiographic assessment should be systematic. We did not perform a 24‐hour post‐run echocardiographic assessment, which would have provided more information on the reversibility of the observed changes in ventricular strain. Objective baseline cardiopulmonary fitness via maximum oxygen uptake (VO_2_max) of study participants was not available, hence, the ACSM guidelines were used as a surrogate for maximum heart rate and subsequent calculation of exercise intensity.

## 5 CONCLUSION

In this study of recreational runners completing a 21 km treadmill run, we observed a reversible elevation of cardiac biomarkers of myocardial injury and hemodynamic stress, which was associated with ventricular stunning, as evidenced by reduced biventricular strain. We further observed changes in markers of renal function consistent with RIFLE stage I AKI, despite a climate controlled indoor environment and full access to oral hydration. These observations suggest transient cardiac and renal perturbations following a 21 km endurance run. With widespread participation of recreational runners in half‐marathon distance running, our findings support further study of the cardiovascular effects of repeated endurance running in the majority of recreational runners, including longitudinal evaluation of long term clinical sequelae associated with the dynamic biochemical and imaging changes observed in this study.

## CONFLICT OF INTEREST

The authors declare no potential conflict of interests.

## AUTHOR CONTRIBUTION

This study was supported by the establishment fund of the Cardiovascular Research Institute, National University Health System, Singapore. MYC receives salary support from Ministry of Health Singapore's National Medical Research Council under its Clinician Scientist‐Investigator scheme (NMRC/CSA‐INV/0006/2016).

## Data Availability

The data that support the findings of this study are available from the corresponding author upon reasonable request.
